# Tetra­kis(1,10-phenanthroline)bis­(μ-pyridine-2,6-dicarboxyl­ato)(pyridine-2,6-di­carboxyl­ato)dicopper(II)terbium(III) nitrate tetra­hydrate

**DOI:** 10.1107/S1600536812031686

**Published:** 2012-07-18

**Authors:** Wei Zhang

**Affiliations:** a Nanyang Medical College, Nanyang 473061, People’s Republic of China

## Abstract

The asymmetric unit of the title compound, [Cu_2_Tb(C_7_H_3_NO_4_)_3_(C_12_H_8_N_2_)_4_]NO_3_·4H_2_O, consists of one-half of the *C*_2_-symmetric trinuclear coordination cation, one-half of the *C*_2_-symmetric nitrate anion and two water mol­ecules. In the coordination cation, the Cu^II^ atom is coordinated by four N atoms from two 1,10-phenanthroline ligands and two O atoms from a bridging–chelating carboxyl­ate group of the pyridine-2,6-dicarboxyl­ate anion, completing a distorted N_4_O_2_ octa­hedral coordination environment. The Tb^III^ atom, located on a twofold rotation axis, is nine-coordinated by three tridentate pyridine-2,6-dicarboxyl­ate anions forming an N_3_O_6_ donor set. The intra­molecular Cu⋯Tb distance of 5.0592 (11) Å indicates weak inter­actions between the Cu^II^ and Tb^III^ atoms. The coordination cations, nitrate anions and water mol­ecules are connected *via* O—H⋯O hydrogen bonds into layers parallel to the (001) plane. Moreover, there are extensive π–π stacking inter­actions [centroid–centroid distances = 4.332 (7) and 3.878 (5) Å] between the phenanthroline ligands and between phenanthroline and pyridine-2,6-dicarboxyl­ate ligands.

## Related literature

For the photophysical properties of lanthanide(III) coordination compounds, see: Jüstel *et al.* (1998 ▶). For the Cu—O, Cu—N, Tb—O and Tb—N bond lengths in previously reported dinuclear copper(II)–terbium(III) coordination compounds, see: Sun *et al.* (2010 ▶); Yang *et al.* (2006 ▶).
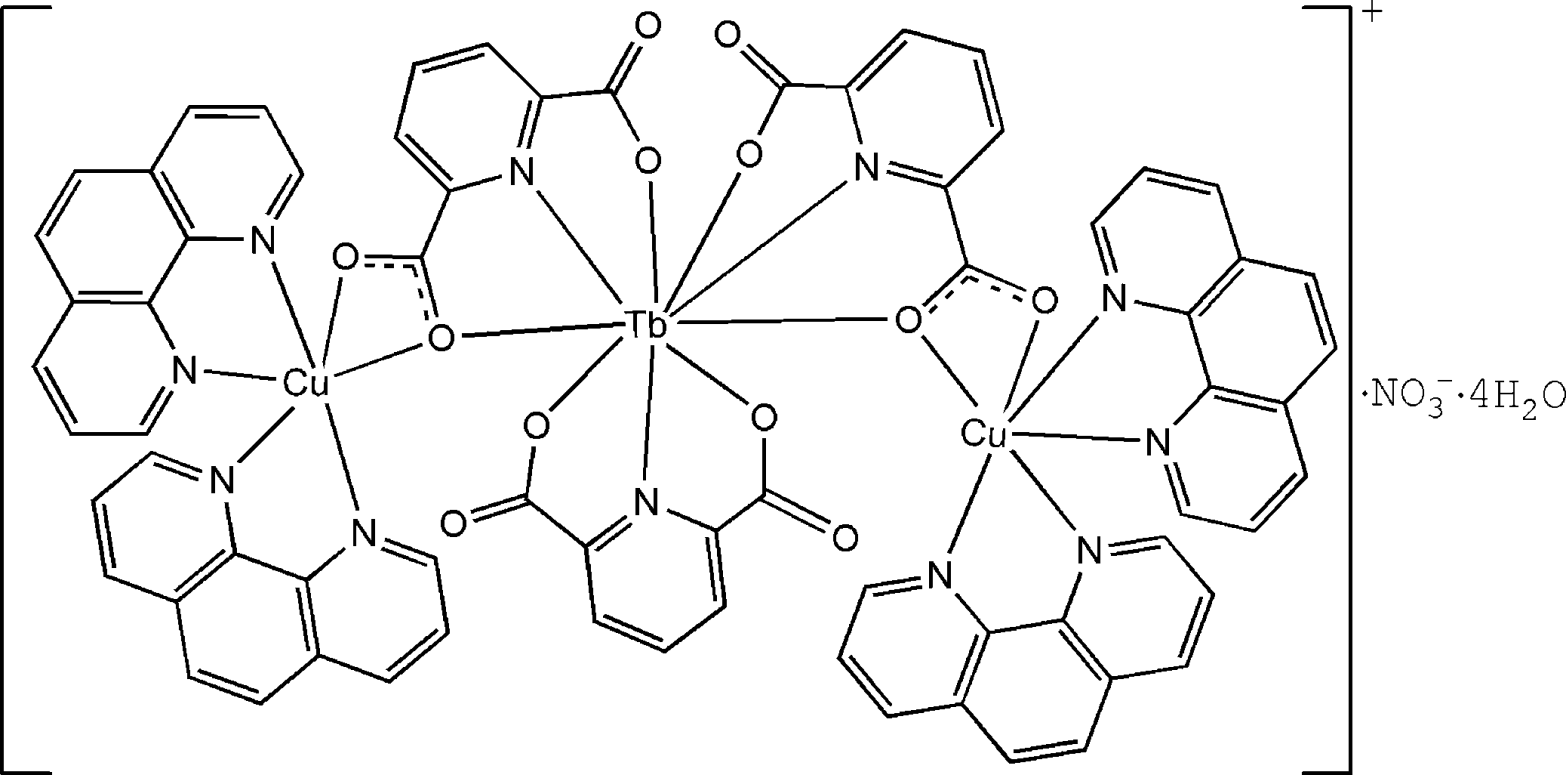


## Experimental

### 

#### Crystal data

[Cu_2_Tb(C_7_H_3_NO_4_)_3_(C_12_H_8_N_2_)_4_]NO_3_·4H_2_O
*M* *_r_* = 1636.20
Monoclinic, 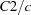

*a* = 17.058 (4) Å
*b* = 19.574 (5) Å
*c* = 19.927 (5) Å
β = 97.289 (4)°
*V* = 6599 (3) Å^3^
*Z* = 4
Mo *K*α radiation
μ = 1.78 mm^−1^
*T* = 296 K
0.19 × 0.17 × 0.15 mm

#### Data collection

Bruker APEXII CCD diffractometer
Absorption correction: multi-scan (*SADABS*; Bruker, 2008 ▶) *T*_min_ = 0.728, *T*_max_ = 0.776
17945 measured reflections
6454 independent reflections
4952 reflections with *I* > 2σ(*I*)
*R*_int_ = 0.029

#### Refinement

*R*[*F* ^2^ > 2σ(*F* ^2^)] = 0.041
*wR*(*F* ^2^) = 0.134
*S* = 1.04
6454 reflections
479 parameters
27 restraints
H atoms treated by a mixture of independent and constrained refinement
Δρ_max_ = 0.88 e Å^−3^
Δρ_min_ = −1.09 e Å^−3^

### 

Data collection: *APEX2* (Bruker, 2008 ▶); cell refinement: *SAINT* (Bruker, 2008 ▶); data reduction: *SAINT*; program(s) used to solve structure: *SHELXS97* (Sheldrick, 2008 ▶); program(s) used to refine structure: *SHELXL97* (Sheldrick, 2008 ▶); molecular graphics: *SHELXTL* (Sheldrick, 2008 ▶); software used to prepare material for publication: *SHELXTL*.

## Supplementary Material

Crystal structure: contains datablock(s) I, global. DOI: 10.1107/S1600536812031686/gk2483sup1.cif

Structure factors: contains datablock(s) I. DOI: 10.1107/S1600536812031686/gk2483Isup2.hkl

Additional supplementary materials:  crystallographic information; 3D view; checkCIF report

## Figures and Tables

**Table 1:** Selected bond lengths (Å)

Tb1—O5	2.374 (3)
Tb1—O3	2.422 (4)
Tb1—N6	2.450 (5)
Tb1—O1	2.489 (3)
Tb1—N5	2.542 (4)
Cu1—N3	2.011 (4)
Cu1—N1	2.019 (4)
Cu1—N2	2.027 (4)
Cu1—O2	2.038 (4)
Cu1—N4	2.195 (4)
Cu1—O1	2.667 (3)

**Table 2:** Hydrogen-bond geometry (Å, °)

*D*—H⋯*A*	*D*—H	H⋯*A*	*D*⋯*A*	*D*—H⋯*A*
O1*W*—H1*WB*⋯O2	0.85 (2)	2.16 (2)	2.979 (7)	162 (4)
O2*W*—H2*WB*⋯O4	0.84 (2)	1.87 (3)	2.705 (9)	174 (11)
O2*W*—H2*WA*⋯O7	0.86 (2)	1.84 (2)	2.675 (14)	164 (10)
O1*W*—H1*WA*⋯O2*W* ^i^	0.91 (2)	1.93 (2)	2.827 (12)	171 (10)

Symmetry code: (i) 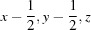.

**Fig. 1.**
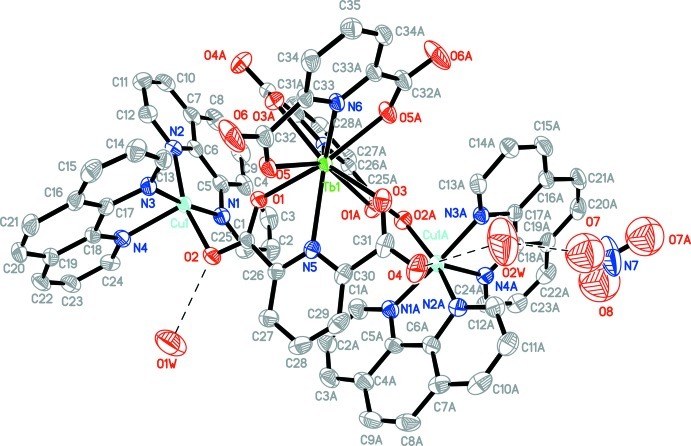
View of the title molecule with displacement ellipsoids drawn at the 30% probability level. H atoms were omitted for clarity. Atoms with the A label are generated by the -x + 1, y, -z + 1/2 symmetry operation.

**Fig. 2.**
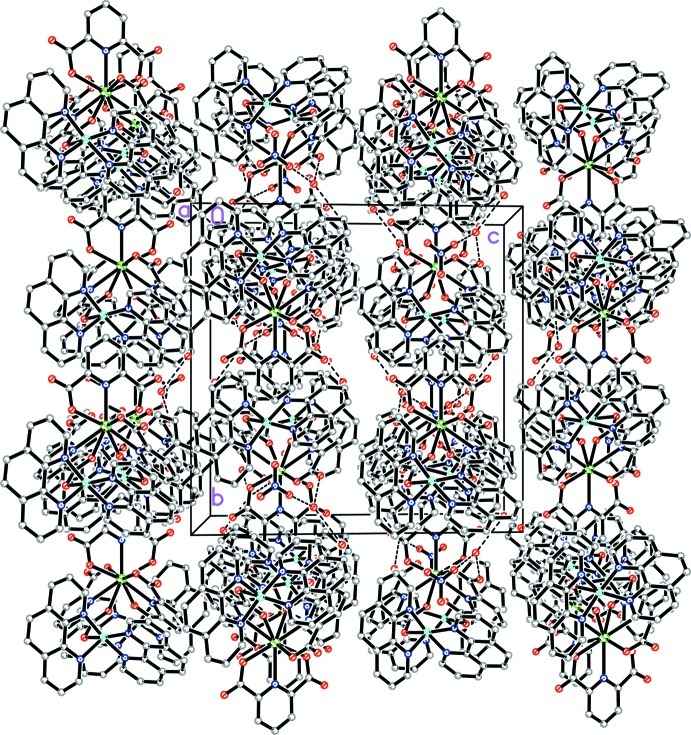
View of the crystal packing along the a axis. For the sake of clarity, H atoms have been omitted.

## supplementary crystallographic information

### Comment

The lanthanide(III) coordination compounds have received much attention in recent years owing to their interesting structures, photophysical properties (Jüstel *et al.*, 1998) and potential applications. In this article, we report the structure of a novel copper(II)–terbium(III) coordination compound obtained by hydrothermal method using the pyridine-2,6-dicarboxylate and 1,10-phenanthroline ligands, {[Cu^II^(C_12_H_8_N_2_)_2_]_2_[Tb^III^(C_7_H_3_NO_4_)_3_]}NO_3_.4H_2_O (Fig. 1). In the coordination cation {[Cu^II^(C_12_H_8_N_2_)_2_]_2_[Tb^III^(C_7_H_3_NO_4_)_3_]}^+^, the Cu^II^ atom is coordinated by four N atoms from two 1,10-phenanthroline ligands and two O atoms from one pyridine-2,6-dicarboxylate completing distorted CuN_4_O_2_ octahedral coordination environment. The Tb^III^ atom located on a two-fold rotation axis is nine-coordinated by three tridentate 2,6-pyridinedicarboxylate anions forming N_3_O_6_ donor set. The shortest distance of Cu···Tb is 5.0592 (11) Å, which indicates there are weak interactions between Cu^II^ and Tb^III^ ions. The details of bond lengths are given in Table 1. These bond lengths of Cu—O, Cu—N, Tb—O and Tb—N type fall in the typical range observed in previously reported copper(II)–terbium(III) coordination compounds (Sun *et al.*, 2010; Yang *et al.*, 2006). The coordination cations {[Cu^II^(C_12_H_8_N_2_)_2_]_2_[Tb^III^(C_7_H_3_NO_4_)_3_]}^+^, nitrate anions and water molecules are connected via O—H···O hydrogen bonds into layered structure parallel to (001) (Fig. 2). In addition, there are extensive π–π stacking interactions between the phenanthroline ligands and between phenanthroline and pyridinedicarboxylate ligands. The hydrogen bonds and π–π stacking interactions play a crucial role in stability of the crystal structure.

### Experimental

All chemicals were of reagent grade quality obtained from commercial sources and used without further purification. The compound was obtained by using hydrothermal method in a 50 ml Teflon-lined autoclave. The mixture of 0.17 g CuCl_2_.2H_2_O, 0.27 g Tb(NO_3_)_3_.6H_2_O, 0.17 g pyridine-2,6-dicarboxylic acid, 0.16 g 1,10-phenanthroline and 20 ml H_2_O was stirred for half an hour, and transferred into a Teflon-lined stainless steel autoclave (50 ml), then treated at 433 K for 6 d. After the mixture was slowly cooled to room temperature, blue block crystals suitable for X-ray structure determination were obtained. The chemical composition of the title compound was confirmed by elemental analysis. The C, H, and N elements analysis were performed on a PerkinElmer 2400II elemental analyzer. Anal. calcd for the title compound: C, 50.65; H, 3.02; N,10.27%. Found: C, 51.22; H, 3.65; N, 9.89%. The results well support the formula of the compound based on the single-crystal X-ray analysis.

### Refinement

The H atoms bonded to C atoms were positioned geometrically and refined using a riding model, with C—H = 0.93 Å, and with *U*_iso_(H) = 1.2 times *U*_eq_(C). The H atoms bonded to O1W and O2W were located in Fourier difference maps and refined with restraints [O—H = 0.83 (2) Å, H···H 1.37 (2) Å]. The H1WA, H1WB and H2WA atoms were refined with additional restraints (SHELXL97 instructions: DFIX 1.85 0.02 H1WA O2W^i^, DFIX 3.60 0.02 H1WB Cu1 and DFIX 1.80 0.02 H2WA O7). In addition, restraints were imposed on the geometry of the nitrate anion and on the dispalcement parameters of its O and N atoms (SHELXL97 instructions: ISOR 0.001 O7 O8, DELU 0.01 N7 O7 N7 O8, DFIX 1.30 0.02 N7 O7 N7 O8). An attempt to refine a disordered model for the nitrate anion was unsuccessful.

### Crystal data

**Table:**

[Cu_2_Tb(C_7_H_3_NO_4_)_3_(C_12_H_8_N_2_)_4_]NO_3_·4H_2_O	*F*(000) = 3288
*M**_r_* = 1636.20	*D*_x_ = 1.647 Mg m^−^^3^
Monoclinic, *C*2/*c*	Mo *K*α radiation, λ = 0.71073 Å
Hall symbol: -C 2yc	Cell parameters from 5232 reflections
*a* = 17.058 (4) Å	θ = 2.3–25.0°
*b* = 19.574 (5) Å	µ = 1.78 mm^−^^1^
*c* = 19.927 (5) Å	*T* = 296 K
β = 97.289 (4)°	Block, blue
*V* = 6599 (3) Å^3^	0.19 × 0.17 × 0.15 mm
*Z* = 4	

### Data collection

**Table:**

Bruker APEXII CCD diffractometer	6454 independent reflections
Radiation source: fine-focus sealed tube	4952 reflections with *I* > 2σ(*I*)
Graphite monochromator	*R*_int_ = 0.029
φ and ω scans	θ_max_ = 26.0°, θ_min_ = 1.8°
Absorption correction: multi-scan (*SADABS*; Bruker, 2008)	*h* = −21→11
*T*_min_ = 0.728, *T*_max_ = 0.776	*k* = −24→22
17945 measured reflections	*l* = −24→24

### Refinement

**Table:**

Refinement on *F*^2^	Primary atom site location: structure-invariant direct methods
Least-squares matrix: full	Secondary atom site location: difference Fourier map
*R*[*F*^2^ > 2σ(*F*^2^)] = 0.041	Hydrogen site location: inferred from neighbouring sites
*wR*(*F*^2^) = 0.134	H atoms treated by a mixture of independent and constrained refinement
*S* = 1.04	*w* = 1/[σ^2^(*F*_o_^2^) + (0.0792*P*)^2^ + 8.9094*P*] where *P* = (*F*_o_^2^ + 2*F*_c_^2^)/3
6454 reflections	(Δ/σ)_max_ = 0.001
479 parameters	Δρ_max_ = 0.88 e Å^−^^3^
27 restraints	Δρ_min_ = −1.09 e Å^−^^3^

### Special details

**Table:**

Geometry. All esds (except the esd in the dihedral angle between two l.s. planes) are estimated using the full covariance matrix. The cell esds are taken into account individually in the estimation of esds in distances, angles and torsion angles; correlations between esds in cell parameters are only used when they are defined by crystal symmetry. An approximate (isotropic) treatment of cell esds is used for estimating esds involving l.s. planes.
Refinement. Refinement of F^2^ against ALL reflections. The weighted R-factor wR and goodness of fit S are based on F^2^, conventional R-factors R are based on F, with F set to zero for negative F^2^. The threshold expression of F^2^ > 2sigma(F^2^) is used only for calculating R-factors(gt) etc. and is not relevant to the choice of reflections for refinement. R-factors based on F^2^ are statistically about twice as large as those based on F, and R-factors based on ALL data will be even larger.

### Fractional atomic coordinates and isotropic or equivalent isotropic displacement parameters (Å^2^)

**Table:**

	*x*	*y*	*z*	*U*_iso_*/*U*_eq_	
Tb1	0.5000	0.828451 (16)	0.2500	0.04016 (14)	
C1	0.3780 (4)	0.5559 (3)	0.2339 (3)	0.0657 (16)	
H1A	0.4057	0.5538	0.2772	0.079*	
C2	0.4024 (4)	0.5136 (3)	0.1828 (4)	0.0766 (19)	
H2A	0.4466	0.4858	0.1921	0.092*	
C3	0.3608 (4)	0.5140 (3)	0.1199 (4)	0.0725 (19)	
H3A	0.3762	0.4862	0.0860	0.087*	
C4	0.2954 (4)	0.5561 (3)	0.1064 (3)	0.0611 (16)	
C5	0.2759 (3)	0.5984 (2)	0.1595 (3)	0.0461 (12)	
C6	0.2115 (3)	0.6447 (3)	0.1487 (3)	0.0488 (12)	
C7	0.1630 (4)	0.6464 (3)	0.0854 (3)	0.0569 (14)	
C8	0.1825 (5)	0.6017 (4)	0.0330 (3)	0.0730 (19)	
H8A	0.1509	0.6013	−0.0086	0.088*	
C9	0.2464 (5)	0.5600 (4)	0.0431 (3)	0.0733 (19)	
H9A	0.2586	0.5330	0.0075	0.088*	
C10	0.0997 (4)	0.6915 (4)	0.0792 (3)	0.0674 (18)	
H10A	0.0657	0.6939	0.0389	0.081*	
C11	0.0872 (4)	0.7326 (3)	0.1327 (3)	0.0668 (16)	
H11A	0.0444	0.7624	0.1290	0.080*	
C12	0.1384 (3)	0.7293 (3)	0.1914 (3)	0.0573 (14)	
H12A	0.1299	0.7587	0.2264	0.069*	
C13	0.2621 (3)	0.8081 (3)	0.3376 (3)	0.0542 (14)	
H13A	0.2924	0.8213	0.3041	0.065*	
C14	0.2405 (4)	0.8578 (3)	0.3827 (3)	0.0639 (16)	
H14A	0.2565	0.9030	0.3795	0.077*	
C15	0.1954 (4)	0.8384 (3)	0.4313 (4)	0.0676 (18)	
H15A	0.1799	0.8708	0.4612	0.081*	
C16	0.1722 (3)	0.7695 (3)	0.4365 (3)	0.0527 (13)	
C17	0.1964 (3)	0.7228 (3)	0.3895 (3)	0.0456 (12)	
C18	0.1765 (3)	0.6513 (3)	0.3928 (3)	0.0445 (11)	
C19	0.1323 (3)	0.6287 (3)	0.4433 (3)	0.0550 (14)	
C20	0.1089 (4)	0.6773 (4)	0.4919 (3)	0.0637 (17)	
H20A	0.0809	0.6624	0.5263	0.076*	
C21	0.1273 (3)	0.7437 (4)	0.4877 (3)	0.0645 (17)	
H21A	0.1105	0.7741	0.5188	0.077*	
C22	0.1136 (4)	0.5585 (3)	0.4430 (3)	0.0642 (16)	
H22A	0.0844	0.5409	0.4754	0.077*	
C23	0.1379 (4)	0.5171 (3)	0.3959 (3)	0.0634 (16)	
H23A	0.1245	0.4710	0.3950	0.076*	
C24	0.1833 (3)	0.5435 (3)	0.3486 (3)	0.0542 (13)	
H24A	0.2011	0.5140	0.3173	0.065*	
C25	0.4261 (3)	0.6997 (2)	0.3338 (3)	0.0398 (11)	
C26	0.5055 (3)	0.7063 (2)	0.3749 (2)	0.0413 (11)	
C27	0.5311 (4)	0.6663 (3)	0.4298 (3)	0.0519 (14)	
H27A	0.4987	0.6326	0.4444	0.062*	
C28	0.6076 (4)	0.6775 (3)	0.4636 (3)	0.0631 (17)	
H28A	0.6269	0.6517	0.5013	0.076*	
C29	0.6531 (3)	0.7277 (3)	0.4394 (3)	0.0608 (16)	
H29A	0.7041	0.7359	0.4605	0.073*	
C30	0.6227 (3)	0.7663 (3)	0.3834 (3)	0.0476 (12)	
C31	0.6681 (3)	0.8235 (3)	0.3539 (3)	0.0556 (15)	
C32	0.4367 (3)	0.9434 (3)	0.3500 (3)	0.0543 (14)	
C33	0.4679 (3)	0.9880 (2)	0.2977 (2)	0.0427 (11)	
C34	0.4668 (3)	1.0585 (3)	0.2987 (3)	0.0540 (14)	
H34A	0.4438	1.0817	0.3319	0.065*	
Cu1	0.27722 (4)	0.66689 (3)	0.28488 (3)	0.04248 (18)	
N1	0.3180 (3)	0.5977 (2)	0.2226 (2)	0.0490 (10)	
N2	0.1994 (3)	0.6868 (2)	0.2016 (2)	0.0475 (10)	
N3	0.2413 (2)	0.7437 (2)	0.3407 (2)	0.0454 (10)	
N4	0.2020 (3)	0.6093 (2)	0.3464 (2)	0.0478 (10)	
N5	0.5497 (2)	0.7559 (2)	0.3524 (2)	0.0411 (9)	
N6	0.5000	0.9536 (3)	0.2500	0.0408 (13)	
N7	1.0000	0.8539 (5)	0.2500	0.118 (4)	
O1	0.41131 (19)	0.73634 (17)	0.28216 (17)	0.0446 (8)	
O1W	0.3730 (5)	0.5423 (4)	0.4498 (4)	0.142 (3)	
H1WA	0.371 (6)	0.5065 (12)	0.4206 (18)	0.170*	
O2	0.3767 (2)	0.65755 (18)	0.35262 (19)	0.0489 (9)	
O2W	0.8497 (6)	0.9270 (4)	0.3641 (7)	0.191 (4)	
O3	0.6303 (2)	0.8570 (2)	0.3059 (2)	0.0581 (10)	
O4	0.7371 (3)	0.8336 (2)	0.3812 (3)	0.0836 (15)	
O5	0.4470 (2)	0.87888 (18)	0.34281 (19)	0.0547 (10)	
O6	0.4060 (4)	0.9707 (2)	0.3959 (3)	0.105 (2)	
C35	0.5000	1.0940 (4)	0.2500	0.056 (2)	
H35	0.5000	1.1415	0.2500	0.067*	
O8	1.0000	0.7900 (9)	0.2500	0.242 (6)	
O7	0.9613 (7)	0.8826 (5)	0.2917 (6)	0.208 (4)	
H2WB	0.814 (6)	0.900 (5)	0.372 (8)	0.250*	
H2WA	0.882 (7)	0.905 (5)	0.343 (8)	0.250*	
H1WB	0.385 (3)	0.575 (2)	0.425 (3)	0.250*	

### Atomic displacement parameters (Å^2^)

**Table:**

	*U*^11^	*U*^22^	*U*^33^	*U*^12^	*U*^13^	*U*^23^
Tb1	0.0415 (2)	0.0376 (2)	0.0439 (2)	0.000	0.01540 (15)	0.000
C1	0.069 (4)	0.053 (3)	0.077 (4)	0.010 (3)	0.014 (3)	−0.003 (3)
C2	0.078 (5)	0.054 (4)	0.101 (6)	0.009 (3)	0.027 (4)	−0.015 (4)
C3	0.085 (5)	0.054 (4)	0.085 (5)	−0.012 (3)	0.036 (4)	−0.021 (3)
C4	0.080 (4)	0.047 (3)	0.061 (4)	−0.019 (3)	0.027 (3)	−0.011 (3)
C5	0.060 (3)	0.037 (3)	0.044 (3)	−0.012 (2)	0.016 (2)	−0.005 (2)
C6	0.059 (3)	0.046 (3)	0.044 (3)	−0.010 (2)	0.018 (2)	0.002 (2)
C7	0.064 (4)	0.060 (3)	0.046 (3)	−0.022 (3)	0.005 (3)	0.006 (3)
C8	0.093 (5)	0.085 (5)	0.042 (3)	−0.034 (4)	0.010 (3)	−0.009 (3)
C9	0.096 (5)	0.073 (4)	0.053 (4)	−0.028 (4)	0.021 (4)	−0.021 (3)
C10	0.062 (4)	0.078 (4)	0.059 (4)	−0.022 (3)	−0.005 (3)	0.024 (3)
C11	0.054 (4)	0.070 (4)	0.074 (4)	0.004 (3)	0.000 (3)	0.015 (3)
C12	0.060 (4)	0.054 (3)	0.058 (4)	0.005 (3)	0.009 (3)	0.003 (3)
C13	0.052 (3)	0.048 (3)	0.066 (4)	0.003 (2)	0.022 (3)	0.002 (3)
C14	0.062 (4)	0.055 (3)	0.077 (4)	0.004 (3)	0.015 (3)	−0.012 (3)
C15	0.065 (4)	0.070 (4)	0.068 (4)	0.021 (3)	0.010 (3)	−0.020 (3)
C16	0.047 (3)	0.065 (4)	0.047 (3)	0.011 (3)	0.007 (2)	−0.007 (3)
C17	0.038 (3)	0.056 (3)	0.044 (3)	0.005 (2)	0.007 (2)	0.000 (2)
C18	0.040 (3)	0.055 (3)	0.037 (3)	−0.001 (2)	0.003 (2)	0.006 (2)
C19	0.041 (3)	0.076 (4)	0.048 (3)	0.003 (3)	0.007 (2)	0.012 (3)
C20	0.050 (3)	0.097 (5)	0.047 (3)	0.006 (3)	0.018 (3)	0.015 (3)
C21	0.060 (4)	0.090 (5)	0.047 (3)	0.018 (3)	0.020 (3)	−0.007 (3)
C22	0.060 (4)	0.076 (4)	0.056 (4)	−0.013 (3)	0.008 (3)	0.027 (3)
C23	0.068 (4)	0.062 (4)	0.059 (4)	−0.010 (3)	0.002 (3)	0.016 (3)
C24	0.060 (3)	0.050 (3)	0.050 (3)	−0.002 (3)	−0.001 (3)	0.007 (3)
C25	0.044 (3)	0.036 (2)	0.042 (3)	0.002 (2)	0.016 (2)	−0.001 (2)
C26	0.045 (3)	0.041 (3)	0.040 (3)	0.008 (2)	0.009 (2)	−0.003 (2)
C27	0.061 (4)	0.052 (3)	0.045 (3)	0.013 (2)	0.010 (3)	0.001 (2)
C28	0.067 (4)	0.072 (4)	0.048 (3)	0.024 (3)	−0.001 (3)	−0.003 (3)
C29	0.048 (3)	0.077 (4)	0.054 (4)	0.014 (3)	−0.007 (3)	−0.017 (3)
C30	0.042 (3)	0.052 (3)	0.049 (3)	0.004 (2)	0.007 (2)	−0.017 (2)
C31	0.043 (3)	0.065 (4)	0.060 (4)	−0.005 (3)	0.009 (3)	−0.025 (3)
C32	0.064 (4)	0.049 (3)	0.056 (3)	0.000 (3)	0.029 (3)	−0.004 (3)
C33	0.045 (3)	0.044 (3)	0.042 (3)	0.001 (2)	0.016 (2)	−0.006 (2)
C34	0.056 (3)	0.045 (3)	0.066 (4)	0.002 (2)	0.024 (3)	−0.010 (3)
Cu1	0.0467 (4)	0.0429 (4)	0.0393 (4)	0.0008 (2)	0.0111 (3)	0.0000 (2)
N1	0.055 (3)	0.041 (2)	0.053 (3)	0.0006 (19)	0.015 (2)	−0.0004 (19)
N2	0.054 (3)	0.048 (2)	0.041 (2)	−0.002 (2)	0.008 (2)	0.0044 (19)
N3	0.046 (2)	0.046 (2)	0.047 (2)	−0.0006 (18)	0.0144 (19)	−0.0005 (19)
N4	0.048 (2)	0.051 (3)	0.045 (2)	−0.0033 (19)	0.0078 (19)	0.005 (2)
N5	0.039 (2)	0.043 (2)	0.042 (2)	0.0025 (17)	0.0087 (18)	−0.0064 (18)
N6	0.043 (3)	0.039 (3)	0.044 (3)	0.000	0.018 (3)	0.000
N7	0.107 (8)	0.069 (6)	0.197 (13)	0.000	0.088 (8)	0.000
O1	0.0407 (18)	0.0471 (19)	0.046 (2)	−0.0036 (14)	0.0055 (15)	0.0081 (16)
O1W	0.189 (7)	0.125 (6)	0.113 (5)	−0.029 (5)	0.026 (5)	0.033 (4)
O2	0.052 (2)	0.046 (2)	0.049 (2)	−0.0056 (16)	0.0104 (17)	0.0090 (16)
O2W	0.183 (9)	0.088 (5)	0.324 (14)	−0.044 (5)	0.122 (9)	−0.032 (6)
O3	0.049 (2)	0.054 (2)	0.071 (3)	−0.0122 (18)	0.011 (2)	−0.009 (2)
O4	0.044 (2)	0.105 (4)	0.099 (4)	−0.019 (2)	−0.003 (2)	−0.013 (3)
O5	0.070 (3)	0.047 (2)	0.054 (2)	0.0024 (17)	0.0351 (19)	0.0037 (17)
O6	0.180 (6)	0.062 (3)	0.094 (4)	0.001 (3)	0.101 (4)	−0.005 (3)
C35	0.063 (5)	0.040 (4)	0.067 (5)	0.000	0.014 (4)	0.000
O8	0.242 (6)	0.241 (6)	0.243 (6)	0.000	0.0316 (14)	0.000
O7	0.208 (4)	0.208 (4)	0.209 (4)	0.0001 (11)	0.0291 (13)	−0.0002 (11)

### Geometric parameters (Å, º)

**Table:**

Tb1—O5	2.374 (3)	C20—C21	1.342 (9)
Tb1—O5^i^	2.374 (3)	C20—H20A	0.9300
Tb1—O3	2.422 (4)	C21—H21A	0.9300
Tb1—O3^i^	2.422 (4)	C22—C23	1.346 (9)
Tb1—N6	2.450 (5)	C22—H22A	0.9300
Tb1—O1	2.489 (3)	C23—C24	1.393 (8)
Tb1—O1^i^	2.489 (3)	C23—H23A	0.9300
Tb1—N5^i^	2.542 (4)	C24—N4	1.328 (7)
Tb1—N5	2.542 (4)	C24—H24A	0.9300
C1—N1	1.308 (7)	C25—O1	1.254 (6)
C1—C2	1.415 (9)	C25—O2	1.269 (6)
C1—H1A	0.9300	C25—C26	1.497 (7)
C2—C3	1.360 (10)	C26—N5	1.340 (6)
C2—H2A	0.9300	C26—C27	1.373 (7)
C3—C4	1.386 (9)	C27—C28	1.408 (9)
C3—H3A	0.9300	C27—H27A	0.9300
C4—C5	1.416 (7)	C28—C29	1.378 (9)
C4—C9	1.424 (9)	C28—H28A	0.9300
C5—N1	1.366 (7)	C29—C30	1.392 (8)
C5—C6	1.419 (8)	C29—H29A	0.9300
C6—N2	1.374 (7)	C30—N5	1.332 (6)
C6—C7	1.418 (8)	C30—C31	1.522 (8)
C7—C10	1.389 (9)	C31—O4	1.248 (7)
C7—C8	1.433 (9)	C31—O3	1.265 (7)
C8—C9	1.356 (10)	C32—O6	1.233 (7)
C8—H8A	0.9300	C32—O5	1.285 (6)
C9—H9A	0.9300	C32—C33	1.507 (7)
C10—C11	1.372 (9)	C33—N6	1.338 (5)
C10—H10A	0.9300	C33—C34	1.380 (7)
C11—C12	1.371 (8)	C34—C35	1.372 (7)
C11—H11A	0.9300	C34—H34A	0.9300
C12—N2	1.328 (7)	Cu1—N3	2.011 (4)
C12—H12A	0.9300	Cu1—N1	2.019 (4)
C13—N3	1.314 (7)	Cu1—N2	2.027 (4)
C13—C14	1.407 (8)	Cu1—O2	2.038 (4)
C13—H13A	0.9300	Cu1—N4	2.195 (4)
C14—C15	1.364 (9)	Cu1—O1	2.667 (3)
C14—H14A	0.9300	N6—C33^i^	1.338 (5)
C15—C16	1.414 (8)	N7—O8	1.250 (15)
C15—H15A	0.9300	N7—O7	1.258 (10)
C16—C17	1.407 (7)	N7—O7^ii^	1.258 (10)
C16—C21	1.441 (8)	O1W—H1WA	0.908 (19)
C17—N3	1.374 (6)	O1W—H1WB	0.85 (2)
C17—C18	1.444 (7)	O2W—H2WB	0.84 (2)
C18—N4	1.351 (7)	O2W—H2WA	0.86 (2)
C18—C19	1.402 (7)	C35—C34^i^	1.372 (7)
C19—C22	1.410 (8)	C35—H35	0.9300
C19—C20	1.450 (9)		
			
O5—Tb1—O5^i^	130.87 (17)	C19—C18—C17	119.3 (5)
O5—Tb1—O3	87.91 (14)	C18—C19—C22	116.6 (6)
O5^i^—Tb1—O3	81.06 (14)	C18—C19—C20	119.4 (5)
O5—Tb1—O3^i^	81.06 (14)	C22—C19—C20	124.1 (5)
O5^i^—Tb1—O3^i^	87.91 (14)	C21—C20—C19	120.4 (5)
O3—Tb1—O3^i^	153.3 (2)	C21—C20—H20A	119.8
O5—Tb1—N6	65.43 (9)	C19—C20—H20A	119.8
O5^i^—Tb1—N6	65.43 (8)	C20—C21—C16	122.2 (6)
O3—Tb1—N6	76.67 (10)	C20—C21—H21A	118.9
O3^i^—Tb1—N6	76.67 (10)	C16—C21—H21A	118.9
O5—Tb1—O1	78.26 (12)	C23—C22—C19	120.0 (5)
O5^i^—Tb1—O1	143.68 (12)	C23—C22—H22A	120.0
O3—Tb1—O1	126.92 (13)	C19—C22—H22A	120.0
O3^i^—Tb1—O1	74.53 (13)	C22—C23—C24	119.6 (6)
N6—Tb1—O1	136.41 (8)	C22—C23—H23A	120.2
O5—Tb1—O1^i^	143.68 (12)	C24—C23—H23A	120.2
O5^i^—Tb1—O1^i^	78.26 (12)	N4—C24—C23	122.7 (6)
O3—Tb1—O1^i^	74.53 (13)	N4—C24—H24A	118.7
O3^i^—Tb1—O1^i^	126.92 (13)	C23—C24—H24A	118.7
N6—Tb1—O1^i^	136.41 (8)	O1—C25—O2	122.9 (5)
O1—Tb1—O1^i^	87.18 (16)	O1—C25—C26	118.2 (4)
O5—Tb1—N5^i^	137.42 (13)	O2—C25—C26	118.9 (4)
O5^i^—Tb1—N5^i^	74.24 (12)	N5—C26—C27	123.0 (5)
O3—Tb1—N5^i^	133.76 (13)	N5—C26—C25	112.9 (4)
O3^i^—Tb1—N5^i^	64.29 (13)	C27—C26—C25	124.1 (5)
N6—Tb1—N5^i^	123.98 (9)	C26—C27—C28	118.2 (6)
O1—Tb1—N5^i^	69.51 (12)	C26—C27—H27A	120.9
O1^i^—Tb1—N5^i^	62.63 (12)	C28—C27—H27A	120.9
O5—Tb1—N5	74.24 (12)	C29—C28—C27	118.3 (6)
O5^i^—Tb1—N5	137.42 (13)	C29—C28—H28A	120.8
O3—Tb1—N5	64.29 (13)	C27—C28—H28A	120.8
O3^i^—Tb1—N5	133.76 (13)	C28—C29—C30	119.9 (5)
N6—Tb1—N5	123.98 (9)	C28—C29—H29A	120.1
O1—Tb1—N5	62.63 (12)	C30—C29—H29A	120.1
O1^i^—Tb1—N5	69.51 (12)	N5—C30—C29	121.3 (5)
N5^i^—Tb1—N5	112.04 (17)	N5—C30—C31	115.1 (5)
N1—C1—C2	122.5 (6)	C29—C30—C31	123.5 (5)
N1—C1—H1A	118.7	O4—C31—O3	127.6 (6)
C2—C1—H1A	118.7	O4—C31—C30	116.5 (6)
C3—C2—C1	119.6 (6)	O3—C31—C30	115.9 (5)
C3—C2—H2A	120.2	O6—C32—O5	125.9 (5)
C1—C2—H2A	120.2	O6—C32—C33	118.8 (5)
C2—C3—C4	119.7 (6)	O5—C32—C33	115.3 (4)
C2—C3—H3A	120.2	N6—C33—C34	121.3 (5)
C4—C3—H3A	120.2	N6—C33—C32	114.3 (4)
C3—C4—C5	117.6 (6)	C34—C33—C32	124.3 (4)
C3—C4—C9	124.8 (6)	C35—C34—C33	119.3 (5)
C5—C4—C9	117.6 (6)	C35—C34—H34A	120.4
N1—C5—C4	122.4 (5)	C33—C34—H34A	120.4
N1—C5—C6	116.9 (4)	N3—Cu1—N1	173.77 (17)
C4—C5—C6	120.7 (5)	N3—Cu1—N2	95.52 (18)
N2—C6—C7	122.8 (5)	N1—Cu1—N2	82.30 (18)
N2—C6—C5	117.0 (5)	N3—Cu1—O2	89.25 (16)
C7—C6—C5	120.2 (5)	N1—Cu1—O2	91.39 (17)
C10—C7—C6	116.9 (6)	N2—Cu1—O2	164.39 (16)
C10—C7—C8	125.0 (6)	N3—Cu1—N4	80.47 (17)
C6—C7—C8	118.1 (6)	N1—Cu1—N4	105.65 (17)
C9—C8—C7	121.0 (6)	N2—Cu1—N4	100.79 (17)
C9—C8—H8A	119.5	O2—Cu1—N4	94.65 (15)
C7—C8—H8A	119.5	C1—N1—C5	118.2 (5)
C8—C9—C4	122.3 (6)	C1—N1—Cu1	129.6 (4)
C8—C9—H9A	118.9	C5—N1—Cu1	112.2 (3)
C4—C9—H9A	118.9	C12—N2—C6	116.7 (5)
C11—C10—C7	119.9 (6)	C12—N2—Cu1	131.6 (4)
C11—C10—H10A	120.0	C6—N2—Cu1	111.6 (4)
C7—C10—H10A	120.0	C13—N3—C17	119.8 (5)
C12—C11—C10	119.4 (6)	C13—N3—Cu1	126.0 (4)
C12—C11—H11A	120.3	C17—N3—Cu1	114.0 (3)
C10—C11—H11A	120.3	C24—N4—C18	117.8 (5)
N2—C12—C11	124.2 (6)	C24—N4—Cu1	132.6 (4)
N2—C12—H12A	117.9	C18—N4—Cu1	109.4 (3)
C11—C12—H12A	117.9	C30—N5—C26	119.2 (4)
N3—C13—C14	122.6 (5)	C30—N5—Tb1	118.9 (3)
N3—C13—H13A	118.7	C26—N5—Tb1	121.9 (3)
C14—C13—H13A	118.7	C33—N6—C33^i^	119.6 (6)
C15—C14—C13	118.6 (6)	C33—N6—Tb1	120.2 (3)
C15—C14—H14A	120.7	C33^i^—N6—Tb1	120.2 (3)
C13—C14—H14A	120.7	O8—N7—O7	116.6 (7)
C14—C15—C16	120.4 (6)	O8—N7—O7^ii^	116.6 (7)
C14—C15—H15A	119.8	O7—N7—O7^ii^	126.9 (14)
C16—C15—H15A	119.8	C25—O1—Tb1	124.1 (3)
C17—C16—C15	117.5 (5)	H1WA—O1W—H1WB	102 (3)
C17—C16—C21	118.0 (5)	C25—O2—Cu1	105.9 (3)
C15—C16—C21	124.4 (5)	H2WB—O2W—H2WA	108 (4)
N3—C17—C16	121.0 (5)	C31—O3—Tb1	125.3 (4)
N3—C17—C18	118.4 (4)	C32—O5—Tb1	124.5 (3)
C16—C17—C18	120.5 (5)	C34^i^—C35—C34	119.2 (7)
N4—C18—C19	123.2 (5)	C34^i^—C35—H35	120.4
N4—C18—C17	117.4 (4)	C34—C35—H35	120.4

Symmetry codes: (i) −*x*+1, *y*, −*z*+1/2; (ii) −*x*+2, *y*, −*z*+1/2.

### Hydrogen-bond geometry (Å, º)

**Table:**

*D*—H···*A*	*D*—H	H···*A*	*D*···*A*	*D*—H···*A*
O1*W*—H1*WB*···O2	0.85 (2)	2.16 (2)	2.979 (7)	162 (4)
O2*W*—H2*WB*···O4	0.84 (2)	1.87 (3)	2.705 (9)	174 (11)
O2*W*—H2*WA*···O7	0.86 (2)	1.84 (2)	2.675 (14)	164 (10)
O1*W*—H1*WA*···O2*W*^iii^	0.91 (2)	1.93 (2)	2.827 (12)	171 (10)

Symmetry code: (iii) *x*−1/2, *y*−1/2, *z*.
